# Pulmonary Kaposi Sarcoma without Respiratory Symptoms and Skin Lesions in an HIV-Naïve Patient: A Case Report and Literature Review

**DOI:** 10.3390/idr14020028

**Published:** 2022-03-25

**Authors:** Cristina Micali, Ylenia Russotto, Alessio Facciolà, Andrea Marino, Benedetto Maurizio Celesia, Eugenia Pistarà, Grazia Caci, Giuseppe Nunnari, Giovanni Francesco Pellicanò, Emmanuele Venanzi Rullo

**Affiliations:** 1 Unit of Infectious Diseases, Department of Clinical and Experimental Medicine, University of Messina, 98124 Messina, Italy; crysmica@gmail.com (C.M.); grazia.caci15@gmail.com (G.C.); giuseppe.nunnari@unime.it (G.N.); evenanzirullo@gmail.com (E.V.R.); 2 Department of Biomedical and Dental Sciences and Morphofunctional Imaging, University of Messina, 98100 Messina, Italy; alessio.facciola@unime.it; 3 Unit of Infectious Diseases, Department of Clinical and Experimental Medicine, University of Catania, 95131 Catania, Italy; andreamarino9103@gmail.com (A.M.); bmcelesia@gmail.com (B.M.C.); eugeniapistara@gmail.com (E.P.); 4 Unit of Infectious Diseases, Department of Adult and Childhood Human Pathology “Gaetano Barresi”, University of Messina, 98124 Messina, Italy; giovanni.pellicano@unime.it

**Keywords:** pulmonary Kaposi sarcoma, HIV, acquired immunodeficiency syndrome, atypical presentations, naïve patient

## Abstract

Kaposi sarcoma (KS) is a multifocal lympho-angioproliferative, mesenchymal low-grade tumor associated with a γ2-herpesvirus, named Kaposi sarcoma-associated virus or human herpesvirus (KSHV/HHV8). The lung is considered a usual anatomical location of KS, despite being infrequent, often in association with extensive mucocutaneous lesions and very uncommonly as an isolated event. We report a case of a pulmonary KS (pKS) in a human immunodeficiency virus (HIV) naïve patient, which was atypical due to a lack of cutaneous involvement and an absence of respiratory symptoms. The pKS was initially identified as a tumoral suspected nodular lesion and only after immunohistochemical analysis was it characterized as KS. Furthermore, the diagnosis of pKS led to the discovery of the HIV-seropositive status of the patient, previously unknown. Our report underlines the importance of considering pKS even without skin lesions and as a first manifestation of HIV infection. We also reviewed literature on the current knowledge about pKS in people living with HIV (PLWH) to underline how one of the most common HIV/acquired immunodeficiency syndrome (AIDS) associated tumors can have a challenging localization and be difficult to recognize.

## 1. Introduction

The achievements of contemporary medicine and the development of new antiretroviral drugs have enabled people to grow old with HIV [1,2]. Contextually, however, among comorbidities, such as diabetes, cardiovascular diseases, chronic kidney disease (CKD), and age-related diseases, cancers have become one of the main burdens for PLWH [3,4,5,6,7]. 

Over time, the incidence of AIDS-defining cancers (ADCs), such as non-Hodgkin lymphoma and KS, and Hodgkin lymphoma, breast, prostate, and colorectal cancer, has decreased [8,9,10,11,12,13]. Otherwise, the incidence of other tumors, such as melanoma, cervix, anal, liver, and lung cancers, has increased [14,15,16,17,18,19,20]. 

Nonetheless, only a low percentage of PLWH undergo any screening test for cancers. PLWH come to the doctor’s attention when they are symptomatic and, unfortunately, often in an advanced stage of cancer [21,22,23]. 

Moreover, neoplasia is often concomitant to opportunistic infections that can occur at the same site, not only poorly affecting the prognosis but also delaying the diagnosis due to the overlapping of symptoms and imaging [24,25]. 

Despite the availability of vaccines for human papillomavirus (HPV) and hepatitis B virus (HBV), most neoplasia associated with HIV infection is led by oncogenic viruses, such as Epstein–Barr virus (EBV), HPV, HBV, hepatitis C virus (HCV), and KSHV/HHV8 [26,27,28,29]. 

KS is a multifocal lympho-angioproliferative, mesenchymal low-grade tumor associated with KSHV, a γ2-herpesvirus, belonging to the family of DNA viruses Herpeseviridae. KSHV is the etiological agent of KS, but it is also pathogenically related to others lymphoproliferative disorders, such as primary effusion lymphoma (PEL), multicentric Castleman’s disease (MCD), diffuse large B cell lymphoma, and germinotropic lymphoproliferative disorder [30,31,32,33,34,35]. 

KS presents as four clinical variants: classic KS, which mainly affects the extremities of elderly patients and generally follows an indolent chronical course; African KS; immunosuppression-associated KS; and AIDS-associated KS [36]. 

The main complications of KS in PLWH, which is remarkable for its morbidity and mortality, include KS inflammatory cytokine syndrome (KICS), an emerging entity characterized by acute respiratory distress syndrome (ARDS), eventually requiring a ventilator and vasopressor support [37,38].

We report a case of pKS in a patient unknown to be seropositive without any cutaneous involvement nor respiratory symptoms. Our literature review identified only 12 other similar cases. Our report underlines the importance of recognizing this localization of KS especially when it presents without skin lesions and as a first manifestation of HIV infection. We performed a literature review on the current knowledge about pKS in PLWH to draw attention to one of the most common HIV/AIDS-associated tumors that can have challenging localization and diagnosis.

### 1.1. Epidemiology

Despite a decline in incidence since the introduction of antiretroviral therapy (ART), KS remains the most common cancer in PLWH in sub-Saharan Africa, where it is still endemic, and causes significant morbidity and mortality [28,39]. The risk of developing KS is estimated to be more than 30 times higher in PLWH than in the general population and PLWH who develop HIV-subtype KS are significantly younger than people who develop the classical and immunosuppression-associated KS subtypes [40,41]. 

The lung is considered a usual anatomical location of KS, despite being infrequent, and is often in association with extensive mucocutaneous disease and very rarely as an isolated event [30,42,43,44]. It is uncommon in women while it occurs in approximately 90% to 95% of homosexual and bisexual men [45]. In some cases, pKS is the initial presentation of HIV infection, with clinical features suggesting pulmonary involvement that are challenging to distinguish from opportunistic pneumoniae [46,47,48]. In some other cases, the simultaneous occurrence of KS and other diseases has been observed, from the most frequently related Castleman syndrome to less frequent comorbidities, such as psoriasis [49,50]. Sometimes, palatal KS is a strong predictor of pKS [48].

### 1.2. Clinical, Radiological, and Endoscopic Features

The clinical, radiological, and endoscopic features of pKS are summarized in Table 1. 

The main presenting symptoms of pKS are cough, dyspnea, and weight loss, but pleuritic pain, hemoptysis, and wheezing can also be present [48,51,52]. Moreover, KS in the lung can be totally asymptomatic [53]. 

The most suggestive radiological findings are dense, nodular, tumor-like opacities and bilateral linear and/or micronodular opacities around the bronchi and vessels [54]. Pleural effusions are also very common. On a chest CT scan, pKS classically presents with bilateral and symmetric ill-defined nodules in a peribronchovascular distribution, also known as flame-shaped lesions or flame sign [55]. Peribronchovascular interstitium and interlobular septa thickening, ground glass opacity (GGO), dilated blood vessels, and strong enhancement of nodules, consolidations, and lymph nodes are common findings of pKS on a chest CT scan [56]. Sometimes, diffuse interstitial infiltrates are observed in patients with KS and concomitant *Pneumocystis jiroveci* pneumoniae (PJP) [57]. To our best knowledge, in the literature, only one case of pKS who presents with complete lung consolidation is reported [58]. 

Bronchoscopy may be useful in the diagnosis of endobronchial lesions, even if KS is often elusive at bronchoscopy. The endobronchial findings are diagnostic in about 50% of cases and although there is a correlation between tracheobronchial and parenchymal disease, the latter can occur without endobronchial lesions [59,60]. This is due to the patchy distribution of microscopic foci of KS throughout the lung parenchyma. In the tracheobronchial tree, KS has a variable appearance, and apart from those lesions that are clearly KS in an already diagnosed patient, biopsy specimens should be taken from all areas of abnormal mucosa [61]. In contrast to the violaceous appearance of the skin and oropharyngeal lesions, endobronchial KS almost always appears erythematous. The endobronchial lesions vary from diffuse confluent hyperemic areas to discrete lesions scattered throughout the tracheobronchial tree [62,63,64,65]. They may present as flat-to-slightly-raised polypoid red to violaceous lesions on the bronchial mucosa [64,65,66,67]. Alveolar hemorrhage is also suggestive of pKS [68].

Although possible, significant iatrogenic bleeding during flexible bronchoscopy is a rare and usually self-limiting event [69].

### 1.3. Therapy

The treatment approach to KS is difficult due to its heterogeneity in terms of localization, extension, and rate of growth. Local or systemic therapy is tailored to patient conditions and KS characteristics [70,71]. 

Local therapy is reserved for patients with minimal cutaneous disease or for nonresponders to systemic therapy who have rapidly progressive disease, and it is considered as palliative therapy. Topical alitretinoin gel, intralesional vinblastine, laser therapy, oral etoposide, cryotherapy with liquid nitrogen, and excisional surgery are valid options [70,71,72]. Local imiquimod or topical 9-cis-retinoid acid can also be used [73]. Recently, electrochemotherapy (ECT) has emerged as an effective treatment based on the combination of chemotherapy and electroporation, which enhances drug uptake into cancer cells [74]. Radiation therapy (RT) represents the best local treatment for both limited and advanced KS. RT has high response rates and is effective as palliation for pain, bleeding, or edema, with more than 90% showing a positive response and 70% entering complete remission and is also useful for preserving cosmetic appearances [75,76,77]. 

In HIV-related KS, ART is the first-line option and often the only available therapy in lower-income countries, such as South Africa, where treatment with ART alone with stavudine, lamivudine, and nevirapine for KS without visceral involvement showed significant improvement in survival [78,79].

To date, no comparative studies on the efficacy of different ART regimens for KS have been conducted. At the beginning of this century, a retrospective double-center study in France compared non-nucleoside reverse transcriptase inhibitor (NNRTI)-based and nucleoside reverse transcriptase inhibitor (NRTI)-based to protease inhibitor (PI)-based regimens and showed that the suppression of HIV replication seemed to be the key factor in KS control, independently of the CD4+ cell count [80]. Moreover, the study concluded that there did not seem to be any differences in terms of clinical and virological outcome between NNRTI- and NRTI- and PI-based regimens in antiretroviral-naive HIV-infected patients with KS. Furthermore, PI- and NNRTI-based regimens appeared to have a similar efficacy regarding the KS outcome [80]. 

Nonetheless, for a long time, PI-based regimens have been considered indispensable in the treatment of KS, alone or in combination therapy. In mice models, the systemic administration of the PI indinavir or saquinavir has been shown to induce regression in the development of angioproliferative KS-like lesions, promoted by primary human KS cells. In addition, PIs reduce basic fibroblast growth factor (bFGF) and vascular endothelial growth factor (VEGF), both of which are involved in angiogenesis, edema, and KS lesion formation [81,82]. In vivo, PI efficacy is related to simultaneous blocking of several pathways in tumoral cells and the activation of others, such as matrix metalloprotease-2, an enzyme that is key to angiogenesis, tumor growth, invasion, and metastasis [83,84]. Furthermore, PI block the secretion of cytokines involved in KS initiation and maintenance [84]. Some studies have observed the direct effects of ritonavir (RTV) on immune cell activation, proliferation, and susceptibility to apoptosis, showing how RTV inhibits the activation and proliferation of primary endothelial cells and decreases the production of tumor necrosis factor alpha (TNF-alpha), IL-6, IL-8, and VEGF, which are all responsible for the development of KS lesions [85,86]. 

To date, no comparative studies on the efficacy of integrase inhibitors (INIs) plus chemotherapy or alone versus protease inhibitors (PIs) have been conducted. In some cases, starting ART with a dolutegravir (DTG)-based regimen led to regression of KS with or without chemotherapy [87,88]. Although Ridolfo et al. [89] reported a case in which there was no relapses after switching from PI to non-nucleoside reverse transcriptase inhibitor (NNRTI) regimens, to date, there have been several reports of KS patients relapsing after switching from PI-based to INI-based regimen therapy and to an NNRTI-based regimen, suggesting a decreased effectiveness regarding viral control of KSHV using INI and NNRTI compared to PI [90,91,92]. 

Despite the large percentage of patients responding to ART treatment, in some cases, KS recurs or worsens under ART [93]. According to a recent study, the immunological markers associated with clinical KS regression after ART treatment seem to be high plasma levels of interleukin (IL)-5 at baseline, whereas increased levels of IL-6 and the chemokine interferon-inducible protein 10 (IP-10) seem to be associated with KS progression [93]. Moreover, high IL-5 and IL-6 plasma levels also seem to be associated with sustained remission while high IP-10 levels seem to be associated with recurrence of KS [94].

Systemic chemotherapy is necessary in cases of no response to ART and/or locally aggressive, symptomatic, rapidly progressive, or disseminated life-threatening KS with visceral involvement. It is the most effective treatment for pKS and is also recommended for the prevention of immune reconstitution inflammatory syndrome (IRIS) [70,73,95,96]. Moreover, there is an improvement in KSHV-specific T cell responses that lead to a significant reduction in the cellular KSHV viral load and effective suppression of KSHV replication using combination therapy compared to ART alone [97]. First-line agents with a high benefit-to-risk ratio include liposomal anthracyclines doxorubicin (PLD) and daunorubicin (DNX), and paclitaxel [70,71]. Furthermore, chemotherapy plus ART showed greater efficacy in reducing the progression of KS compared to ART alone, without any difference between PLD, DNX, and paclitaxel chemotherapy regimens [96]. DNX is safe and active, leading to complete or partial resolution of baseline pulmonary symptoms, complete or partial improvement in the diffusing capacity of carbon monoxide (DLCO), and complete or partial resolution of radiographic abnormalities [95]. Similar clinical improvements in cough and dyspnea, increase in the arterial oxygen pressure (pO2), and improvement in the radiographic pKS pattern are observed after PLD treatment [98]. Different studies have shown that PLD has a higher response rate on pKS than the bleomycin and vincristine (BV) or doxorubicin, bleomycin, and vincristine (ABV) combination treatments [99,100]. However, even well-tolerated anthracycline-based treatment is more myelosuppressive as the most common treatment-related toxicity of PLD, DNX, and paclitaxel is in fact neutropenia [95,99,101].

### 1.4. Complications

Besides the pulmonary manifestations of pKS, including airway lesions, peribronchovascular opacities, and characteristic hemorrhagic pleural effusions, uncommon complications of pKS include chylothorax, IRIS, and diffuse alveolar hemorrhage (DAH) [102]. 

Chylothorax is a rare cause of pleural effusion, characterized by the pleural fluid having a milky white appearance due to its high levels of cholesterol and triglyceride [103,104]. Chylothorax seldom occurs in patients with pKS, probably because of lymphatic obstruction [105,106]. In most cases reported in the literature, chylothorax presents bilaterally and monoliteral presentation is less common [107,108,109,110,111,112,113]. Pericardial involvement is infrequent; however, development into the chylopericardium has been reported in one case [102,114]. 

Untreated chylothorax has a high morbidity and mortality [115]. The management of pKS-related chylothorax is based on conservative treatments, such as percutaneous chest drainage and dietary measures [115]. When conservative treatments fail, it is necessary to perform other procedures, such as talc pleurodesis or pleuroperitoneal shunt [105,107,116]. Nonetheless, rapidly recurrent chylous effusions are often resistant to conservative treatment and many need surgical intervention [117]. 

IRIS is syndrome of aberrant reconstituted immunity beginning with a rapid normalization of the CD4+ cell count, resulting in a dysregulated immune response [118]. Generally, the onset is between 1 and 22 weeks after starting ART and is usually in the first 12 weeks [119]. The incidence and mortality are higher in sub-Saharan Africa than in the UK, mostly because of the more advanced KS at diagnosis and reduced chemotherapy availability [120]. Moreover, morbidity and mortality are higher in IRIS with visceral KS in comparison to cutaneous KS and to IRIS due to other infections [121]. 

Predictors of the development of IRIS in KS patients are still not well defined. Lung involvement is one predictor [122]. Likewise, measurement of the KSHV viral load in plasma may be useful for the identification of KS patients at risk of IRIS because they have high KSHV load plasma levels [123]. In a prospective study in Mozambique, beside detectable plasma KSHV DNA, another three independent IRIS predictors were identified: clinical pretreatment of KS, hematocrit <30%, and high pre-ART plasma HIV-1 RNA viral load [124]. Moreover, concomitant or recent use of glucocorticoids may contribute to an increased risk of IRIS [125].

Typically, IRIS presents with the unmasking of covert infections or the worsening of overt diseases [126]. Depending on the location of the lesions, IRIS can include a large range of clinical aspects from functional or aesthetic manifestations to a life-threatening situation. Pulmonary localizations are characterized by cough, dyspnea, hemoptysis, parenchymal nodular lesions, adenopathy, and pleural effusions [116]. Severe clinical presentations are characterized by an abrupt fever, thrombocytopenia, anemia, hyponatremia, and hypoalbuminemia [127]. The magnitude of endobronchial lesions can lead to acute airway obstruction that can evolve into a life-threatening situation [127]. 

Moreover, Dumic et al. [127] reported a fatal case of IRIS in a patient with disseminated pKS and cutaneous KS, characterized by the development of rapid clinical deterioration with progressive multiorgan failure, named KICS. 

KICS is an emerging and sometimes difficult to recognize cause of morbidity and mortality. KICS is an MCD-like systemic inflammation, characterized by the classical signs and symptoms of MCD in the absence of MCD-related pathologic evidence [128]. A common feature of MCD and KICS is the overproduction of both IL-6 and IL-10 [129]. Although the underlying pathophysiologic mechanisms of KICS remain unclear, it seems that the cytokine-induced proinflammatory state caused by KSHV in the lytic phase is responsible for the clinical manifestations [128,129]. Clinically, KICS is severe but generally not specific and shares overlapping clinical features, such as lymphadenopathy, cytopenia, and inflammatory symptoms, with MCD and IRIS, which are the main differential diagnoses [38,130]. The main signs and symptoms are fever, sweats, fatigue, wasting, hypoalbuminemia, and hyponatremia, which in some cases are associated with effusions [130]. The distinction between KICS and IRIS may be difficult to determine; however, there are clinical and laboratory differences that can help in the distinction, as reported in Table 2. 

Firstly, in general, KICS has no temporal correlation with ART initiation [38]. From a laboratory point of view, during KICS, a low CD4+ cell count (<100 cells/mmc) and high HIV viral load plasma levels are detected. Otherwise, during IRIS, a vertiginous increase in the CD4+ cell count and low HIV viral load plasma levels are typical [128]. Furthermore, whereas during an IRIS, KSHV viral load plasma levels tend to decrease compared to pre-ART values, at the beginning of KICS symptoms, KSHV viral load plasma levels are usually high [128]. However, the diagnosis of KICS is usually clinical and requires exclusion of MCD [129].

In sub-Saharan Africa and South America, the coadministration of ART and chemotherapy has reduced the incidence not only of early progression of KS but also IRIS associated with KS compared with ART alone [124,131]. In one case, the use of short term corticosteroids, in addition to PLD and ART, resulted in the survival of patients with pKS and worsening of pulmonary lesions up to stenosis of the respiratory tract during IRIS [132].

DAH is a complication of untreated pKS, which is itself an independent risk factor for DAH [43,133]. It is characterized by the clinical findings of dyspnea, cough, hypoxemia, and anemia accompanied by bilateral infiltrates on a chest radiograph or CT scan. The diagnosis is confirmed by bronchoscopic inspection to exclude a focal source of the bleeding [102].

## 2. Case Presentation

We report the case of a man, Caucasian, 47 years old, with no significant comorbidities. In August 2021, after the second shot with the mRNA vaccine for SARS-CoV-2, the man started to manifest recidivant fever, which was resistant to treatment, and started noticing weight loss. Until the first hospitalization 1 month later, the case had lost 13 kg. During the first hospitalization, a chest CT scan was performed, and a solitary nodular formation was found in the upper lobe of the right lung, with thickening that was represented more in the right lung, suggesting an interstitiopathy of uncertain nature. No respiratory symptoms were reported. After dismissal, he was admitted to the thoracic surgery unit for further investigations. 

Physical examination of the thorax was normal except for a pulmonary dullness on the right upper area; no superficial lymphadenopathy of latero-cervicals, retronucals, submandibulars, submentals, supraclavicles, or axillary lymph nodes were found. No appreciable skin lesions were observed. The man was cooperative but showed a general slowness and was partially oriented when he was asked questions. A chest abdomen and brain CT scan was performed, with the following results: solid lesion with spiked margins in the upper right lobe (28 × 19 mm), a consolidation thickening in the right lung, and a general thickening in the interstitium in both lungs with ground glass and crazy paving areas that was represented more in the right lung. No abnormalities were reported in the abdomen. In the brain, an abnormal venous course in the fronto-parietal area was visible. 

With the suspicion of lung cancer, on the 10th day of hospitalization, the man underwent subtotal lobectomy of the right upper lobe. Biopsy of the lung lesions performed by thorax surgeons showed a proliferation of spindle cells with a slit-like vasculature and erythrocyte exudation. Immunohistochemical analysis showed staining of CD31+, CD34+, D2-40, and HHV8, suggesting the presence of KS, with an MIB 1 of 40%. KS was stadiated and oral and pharyngo-tonsillar lesions, histologically and immunohistochemically characterized as KS, were also found. 

In early October, an HIV test was performed and the result was positive, as confirmed by Western blot. The man was a late presenter (CD4+ count < 350/mmc/AIDS defining illness regardless of the CD4+ cell count at HIV diagnosis) with an HIV RNA viral load in plasma of 650,000 cps/mL, accompanied by CD4+ count of 9/mmc (2.4%), CD8+ count of 347.2/mmc (54.2%), and CD4+/CD8+ ratio of 0.04. Clinically, the patient complained of asthenia and denied any respiratory symptoms. ART was started with DTG and emtricitabine (FTC)/tenofovir alafenamide (TAF) without chemotherapy. After 1 month, we observed a reduction in the HIV RNA viral load in plasma of 170 cps/mL, accompanied by an increase in the CD4+ count of 27.7/mmc (5.5%), CD8+ count of 245.5/mmc (50.5%), and CD4+/CD8+ ratio of 0.10. The ART was sufficient to stop the KS progression in the oral and pharyngo-tonsillar region. The rhino-laryngoscopy at the 1-month follow-up showed no lesions. To date, no further lesions have developed.

## 3. Discussion

KS is still one of the most common ADCs [134]. While muco-cutaneous localization is the most recognizable one, there are many other usual localizations of KS, including the lungs, which is sometimes challenging to diagnose [30]. The case we observed is atypical because of the pulmonary localization in the total absence of respiratory symptoms and without any skin lesions suggestive of KS. 

In our case, pKS was found as an incidental report in the first thorax CT scan, when it was not known that the patient was HIV positive, and, before immunohistochemical analysis revealed it was pKS, clinicians thought it could be pulmonary carcinoma. 

In the literature, we found 12 cases of pKS in PLWH similar to ours, with atypical presentation without muco-cutaneous involvement, the main features of which are shown in Table 3 [43,44,62,63,64,65,66,135,136,137,138,139]. 

In six cases, the patients were already known to be HIV positive, with insufficient adherence to ART or who had experienced a short period of time under treatment [43,44,62,63,66,137]. In the other cases and our case, a diagnosis of pKS was made and only consequently was the diagnosis of HIV infection and AIDS made [64,135,136,139].

Eleven patients presented due to various clinical manifestations of pKS, most of them involving the pulmonary system, such as a nonproductive cough, dyspnea, and pleuritic pain. Furthermore, in three cases, hemoptysis was reported as an initial symptom [62,66,137]. 

The case reported by Young et al. [65] is the most similar to ours due to the normality of the pulmonary functional texts; however, this case differs because of the absence of abnormalities on the chest CT scan. The only symptoms our patient complained of were deep asthenia and significant weight loss (about 13 kg) in 1 month, which is similar to 6 of the 12 cases reported in which patients, among other symptoms, experienced weight loss in a short time [43,44,62,63,65,136].

With the only exception of Young et al. [65], in all the other cases, the lesions of pKS were radiologically identified and pKS presented with various features on the chest X-ray and/or chest CT scan. In our patient, the pulmonary lesion was found on the chest CT scan in the right superior lobe as a single nodular solid formation, which was irregular, and then was surgically removed with sub-lobectomy. In the other six cases, pKS presented as a nodular lesion, irregular and solitary or bilateral and multiple [43,62,63,135,138,139]. Beside the nodular formation, in our patient’s chest CT scan, there was also a general thickening in the interstitium, the presence of interstitial infiltrates, and areas of consolidation. The same radiological features are reported in the literature and are quite characteristic of pKS [43,44,64,66,137,138]. 

Chemotherapy was not performed in our patient since the lesion was successfully removed. After the beginning of ART, no IRIS onset nor other KS lesions developed. Notably, in five cases, pKS was treated with ART without chemotherapy, due to the refusal of the patient and his compromised status, as was unable to tolerate chemotherapy [62,64,66,135,136,137]. In the others, chemotherapy was mostly with PLD, with paclitaxel in one case, and with ABV only in one case [43,44,63,65,138]. As in our patient, upper pulmonary lobectomy was performed in one case due to the presence of an irregular solitary nodule [137]. 

Despite the differences in treatments, in six cases, lesions improved and pKS stopped its progression [44,63,65,66,136,138]. On the contrary, the rapid decline in respiratory function resulted in the death of patients in the other cases, confirming the high mortality of this neoplasia [43,62,64,135].

## 4. Conclusions

The case we observed is peculiar not only because of the lack of typical skin lesions recognizable for KS but also due to the absence of respiratory symptoms. Although the lung is considered one of the common localizations of visceral KS, it is challenging to recognize it when it presents in an atypical manner. Considering the high mortality of pKS, consideration of potential visceral involvement of KS in patients newly diagnosed with HIV infection, even without muco-cutaneous involvement and specific signs and symptoms of organ compromission, is of the utmost importance.

## Figures and Tables

**Table 1:** Clinical, radiological, and endobronchial findings of pKS.

Features of Pulmonary Kaposi Sarcoma	
Presenting symptoms	cough
	dyspnea
	weight loss
	pleuritic pain
	hemoptysis
	wheezing
	totally asymptomatic (in some cases)
Radiological findings	pleural effusions
	flame-shaped lesions or flame sign
	interlobular septa thickening
	ground glass opacity (GGO)
	dilated blood vessel
	nodules
	consolidations
	tumor-like opacities
	bilateral linear and/or micronodular opacities around the bronchi and vessels
Endobronchial findings	diffuse confluent hyperemic areas
	discrete lesions scattered throughout the tracheobronchial tree
	flat-to-slightly-raised polypoid red to violaceous lesions on the bronchial mucosa
	alveolar hemorrhage

**Table 2:** Clinical and laboratory differences between KS KICS and IRIS in PLWH.

Differences between KS KICS and IRIS		
	KICS	IRIS
Definition	Clinically MCD in the absence of pathological signs of MCD in PLWH	syndrome of aberrant reconstituted immunity due to rapid normalization of the CD4+ cell count, resulting in a dysregulated immune response
Temporal correlation with ART beginning	None	Within 12 weeks
Clinical manifestations	Fever, sweats, fatigue, wasting, lymphadenopathy, cytopenia, hypoalbuminemia, hyponatremia	unmasking of covert infections or the worsening of overt diseases
KSHV viral load	Is usually high at the beginning of symptoms	tends to decrease compared with pre-ART values
HIV viral load	High	Low
CD4+ cell count	<100/mmc	vertiginous increase in the CD4+ cell count
Cytokine pattern	IL-6 and IL-10	not well characterized to date

Clinical and laboratory differences between KS KICS and IRIS in PLWH. Adapted from Cantos VD et al. [128].

**Table 3:** Main features of the to date reported cases of pKS without respiratory symptoms or skin lesions.

Features of the to Date Reported Cases of pKS without Skin Lesions and/or Respiratory Symptoms							
Authors	Symptoms	On ART	X-rays/CT Scan	Bronchoscopy	Skin Lesions	Treatment	Outcome
Nguyen et al. [43]	Dyspnea Cough Weight loss	Off ART for 2 years	multiple bilateral peribrochovascular nodules	unrevealing	None	ART + PLD	Exitus
Khan et al. [44]	Chest pain Dyspnea Cough Weight loss	No	bilateral lower lung interstitial infiltrates and mild perihilar infiltrates	unrevealing	Yes	ART + PLD	Recovery
Nwabudike et al. [62]	Chest pain Dyspnea Hemoptysis Weight loss	Yes	diffuse fine non-calcified nodular densities in both lungs	Erythematous lesions on the proximal and distal part of trachea and right middle lobe	None	ART	Exitus
Ramos et al. [63]	Dyspnea Purulent sputum Weight loss	Off ART for about 3 years	multiple peribronchovascular nodular lesions	Erythematous focal, red and purple flat mucosa through the primary and segmental bronchi	None	ART + PLD	Recovery
Diaz et al. [64]	No respiratory symptoms	No	extensive bilateral lobar consolidations	Violaceous slightly raised lesions at the junction of the trachea and the entrance to the left main bronchus	None	ART	Exitus
Young et al. [65]	Dyspnea Dry cough Weight loss	-	normal	Diffuse mucosal erythema and friability with cherry-red lesions scattered throughout the tracheobronchial tree	None	Paclitaxel	Recovery
Aboulafia [66]	Dyspnea Hemoptysis	Refused	bilateral scattered parenchymal lesions with some lung consolidations	Numerous and widespread nodular and violaceous lesions	None	ART	Recovery
Imran et al. [135]	Dyspnea Dry cough	No	nodular opacities in both lung bases	unrevealing	None	ART	Exitus
Dirweesh et al. [136]	Productive cough Weight loss	No	right-sided large perihilar mass with multiple bony methastasis	unrevealing	None	ART	Recovery
Roux et al. [137]	Cough Dyspnea Hemoptysis Fever	Yes	dense upper left lobe lesion	unrevealing	None	Surgery	Exitus for septic shoc
Grocín et al. [138]	Fever Cough Dyspnea	-	perihilar interstitial pattern which evolved to bilateral nodular pattern	Presence of KS lesions	No	ABV	Recovery
Romeu et al. [139]	No respiratory symptoms	-	bilateral nodular lesions	-	Yes, but later	-	-

## Data Availability

Not applicable.
